# Factors Influencing Participation in COVID-19 Clinical Trials: A Multi-National Study

**DOI:** 10.3389/fmed.2021.608959

**Published:** 2021-02-23

**Authors:** Ahmed Samir Abdelhafiz, Samar Abd ElHafeez, Mohammad Adnan Khalil, Manal Shahrouri, Bandar Alosaimi, Raneem O. Salem, Mohamed Alorabi, Fatma Abdelgawad, Mamoun Ahram

**Affiliations:** ^1^Department of Clinical Pathology, National Cancer Institute, Cairo University, Cairo, Egypt; ^2^Epidemiology Department, High Institute of Public Health, Alexandria University, Alexandria, Egypt; ^3^Department of Basic Medical Sciences, Faculty of Medicine, King Fahad Medical City, Riyadh, Saudi Arabia; ^4^Office of Monitoring, Research, Learning and Evaluation, Tetra Tech DPK, Amman, Jordan; ^5^Department of Research Labs, Research Center, King Fahad Medical City, Riyadh, Saudi Arabia; ^6^Department of Clinical Oncology, Faculty of Medicine, Ain Shams University, Cairo, Egypt; ^7^Department of Pediatric Dentistry and Dental Public Health, Faculty of Dentistry, Cairo University, Cairo, Egypt; ^8^Department of Physiology and Biochemistry, The University of Jordan, Amman, Jordan

**Keywords:** COVID-19, clinical trials, Arabs, bioethics, attitude

## Abstract

In 2020, the World Health Organization has characterized COVID-19, a disease caused by infection with the SARS-CoV-2 virus, as a pandemic. Although a few vaccines and drugs have been approved to, respectively, prevent or treat the disease, several clinical trials are still ongoing to test new vaccines or drugs to mitigate the burden of the pandemic. Few studies have shown the role of host genetics in disease prognosis and drug response highlighting the importance of diverse participation in COVID-19 clinical trials. The goal of this study is to assess public attitudes in Egypt, Saudi Arabia, and Jordan toward participating in COVID-19 clinical trials and to identify the factors that may influence their attitude. An online questionnaire was developed and distributed among the target group through social media platforms. The number of responses was 1,576. Three quarters (74.9%) of participants heard about clinical trials before, 57.6% of them had a positive attitude toward participation in COVID-19 clinical trials. The conduct of clinical trials in accordance with the scientific, research, and ethical guidelines was a strong predictor of willingness to participate in clinical trials. Other positive factors also included protection of family from COVID-19 and contributing to the return to normal community life as well as receiving additional healthcare benefit was the fourth significant predictor. On the other hand, the thought that clinical trials can have a negative impact on the health of participants strongly predicted the unwillingness of individuals to participate in such trials. This was followed by having limited information about the novel coronavirus and COVID-19 and the lack of trust in physicians and hospitals. In general, Arab citizens are accepting the concept and have a positive attitude toward COVID-19 clinical trials. Increasing awareness of COVID-19 and clinical trials, enforcing the concept of altruism, and placing clear policies in conducting clinical trials are needed to increase participation in clinical trials among Arabs.

## Introduction

In December 2019, the coronavirus disease-19 (COVID-19) was first identified during an outbreak of respiratory illness in Wuhan, China (1). On the 11th of March 2020, the World Health Organization (WHO) declared COVID-19 as a pandemic (2). The disease is caused by the severe acute respiratory syndrome coronavirus 2 (SARS-CoV-2) and is associated with a variety of symptoms ranging from mild, self-limiting respiratory symptoms, to severe, debilitating illness leading to progressive pneumonia, development of cytokine storm, multi-organ failure, or even death (3, 4). Older age, male gender, and the presence of comorbidities were the main risk factors leading to severe complications and death (5, 6). The disease has spread rapidly affecting millions of people around the world, including Arab countries.

COVID-19 is considered a health crisis to individuals. It has impacted and overburdened healthcare systems. Countries have been racing to slow the spread of the virus by testing and treating patients, carrying out contact tracing, limiting travel, quarantining citizens, and canceling large gatherings such as sporting events, concerts, and schools. By stressing every one of the countries it inflicts, it has created devastating social, economic, and political crises that are expected to persist (7). Although some vaccines and drugs have been approved to prevent or treat the disease (8–10), clinical trials are still ongoing to test newer ones that can mitigate the burden of the pandemic. This is particularly important in the case of vaccines considering the insufficient supplies to achieve global immunization against COVID-19. Prevention and treatment of COVID-19 have emerged as critical needs and challenges to find new approaches, which may help in controlling the spread of the pandemic, treating the disease, or alleviate its symptoms. Ethnic variation in the distribution of COVID-19 has been thought to be genetically influenced (11, 12), particularly that certain genetic variants are associated with the clinical outcome of COVID-19 (13, 14). Similarly, the role of genetics in treatment efficacy is also proposed (15). Thus, the participation of various ethnic groups in COVID-19 clinical trials is critical in order to assess the efficacy of treatments.

Willingness to participate in clinical trials could be influenced by several factors including anticipated benefits, patients' understanding of trials, and the level of trust patients place in investigators. In addition, the majority of participants in clinical trials are reluctant to do additional monitoring tests, particularly invasive ones, since they can be associated with potential morbidity or may be inconvenient for the participant (16).

Up to January 2nd of 2021, more than 4,000 clinical trials have been registered for COVID-19. Of them, 154 studies are held in Egypt, 25 studies are conducted in KSA, and nine studies are registered in Jordan (17). While there are structural and demographic challenges for the successful conduct of clinical trials in the Arab region, little is known about perceptions of the public toward participation in clinical trials to prevent or manage COVID-19. Therefore, the current study is conducted to assess the knowledge, attitudes, and perceptions of the general population in, Egypt, KSA, and Jordan toward participation in clinical trials, and to determine the associated factors that may influence their attitude toward participation in COVID-19 clinical trials. The three countries represent different regions of the Arab countries with Egypt representing countries of Northern Africa and Sudan, KSA representing Gulf countries, and Jordan representing the Levant.

## Subjects and Methods

### Study Design and Populations

This is a cross-sectional study that was conducted through an online survey using Google Forms between July 27 and August 4, 2020, in Egypt, KSA, and Jordan. The survey was distributed using different social media platforms according to what is commonly used in each country. Whereas, Twitter was used in Saudi Arabia, Facebook, LinkedIn, and WhatsApp were used in Egypt and Jordan. The authors posted the survey links on their own social media profiles, sent messages to different groups, and asked their contacts to circulate them. Advertisements were also purchased to recruit participants in Egypt reaching over 100,000 individuals. The target audience was adults 18 years and older of both gender, educational background, and economic status. Participants completed the survey after reading a well-developed informed consent that explained the following: purpose and nature of the study, the difference between a drug and a vaccine, the definitions of COVID-19 and clinical trials, and how clinical trials are reviewed and conducted including ethical considerations. The informed consent assured participants of protecting their privacy and confidentiality as anonymity was mentioned explicitly and confirming that collected responses would be analyzed collectively. In addition, participants were assured that the only purpose of their participation was to examine their perceptions and attitudes toward COVID-19 clinical trials, and not to register them for an actual clinical trial. Finally, participants were informed that their participation was voluntary and no financial compensation would be provided. The study protocol was approved by three independent ethics committees: Institutional Review Board, National Cancer Institute, Cairo University, Institutional Review Board, King Fahad Medical City, and Institutional Review Board, Jordan University Hospital, The University of Jordan.

### Measurement

A pre-designed data collection questionnaire was prepared in Arabic and divided into seven sections: basic socio-demographic background (section 1), health status including if they were diagnosed with COVID-19, had suspected to have had COVID-19, or had been in contact with a COVID-19 patient, in addition to a question regarding their diagnosis of a chronic disease(s) and nature of the chronic diseases (section 2), knowledge of clinical trials (yes/no) and, if knowledgeable, sources of this knowledge (section 3), perceptions toward COVID-19 (13 statements with three options of “agree,” “disagree,” and “unsure”) (section 4), motivating factors toward participation in COVID-19 clinical trials (seven statements with three choices: “yes,” “no,” or “unsure”) (section 5), deterring factors of participation in a COVID-19 clinical trial of (14 statements with three choices: “yes,” “no,” or “unsure”) (section 6), and, finally, attitude toward self-participation or participation of a family member in COVID-19 vaccine or drug clinical trials measured by four questions with responses based on a five-point Likert scale of “definitely yes,” “probably yes,” “unsure,” “probably no,” and “definitely no” (section 7). All “unsure” responses were grouped with “no” responses (sections 3–6), and “disagree” responses (section 4). The attitude questions in section 7 were scored as one point for: “definitely yes,” two points for “probably yes” three points for “unsure,” four points for “probably no,” and five points for “definitely no.” Participants who had a sum of 10 or less were considered as having a positive attitude and those with scores of more than 10 were considered to have a negative attitude.

### Psychometric Properties of the Questionnaire

Questionnaire items were formulated in Arabic and verified by all authors who are native Arabic speakers. English translation was put forth for the manuscript purposes only and verified by three of the authors (ASA, MAK, and MA2).

### Pilot Study and Validation

Questionnaire validity was tested using the two-tier verification model. First, 100 participants were recruited (60 from Egypt, 30 from KSA, and 10 from Jordan) and feedback was collected from respondents and discussed by the authors. Unclear or conflicting items were modified to eliminate ambiguity. The questionnaire was re-distributed to 50% of original respondents from the three countries, respectively, at least a week later.

#### Content Validity

Content validity was assessed by an expert panel of five investigators with knowledge and expertise in instrument development. The content clarity was determined for all items. Convergent validity was assessed by calculating item-total correlations for each construct of the questionnaire. Divergent validity was assessed by testing the correlation between total scores for each construct (18).

#### Reliability

The intra-class correlation coefficient (ICC) was used for the assessment of the test-retest reliability, while Cronbach's α coefficient was used to assess the internal consistency of the questionnaire (19).

### Statistical Analysis

Psychometric evaluation of the pilot questionnaire was done by assessment of intra-class correlation coefficient (ICC). Cronbach's α coefficient was also used to assess the internal consistency of the questionnaire. Pearson's correlation analysis was used to calculate item-total and correlation between total scores. Data of the final version of the questionnaire were summarized as frequencies and percentages. Attitudes were classified as either positive or negative as described earlier. Cross-tabulation of categorical data by attitude (positive vs. negative) was done by testing the association using Chi-square. Spearman's correlation coefficient was calculated between the total attitude scores, which were calculated as described earlier, and all variables. Multiple logistic regression model using stepwise approach was constructed for identifying the independent predictors of attitudes toward participation in clinical trials of vaccine or drug treatment of COVID-19. All variables with *P* < 0.05 in the bivariate analysis were included in the model. The final model included gender, the conduct of clinical trials will be in accordance with the scientific research and ethical guidelines, contributing to the protection of my family from COVID-19, receiving additional healthcare benefits, contributing to the protection of my community from COVID-19, the possibility of getting ill prevents me from participating in such trials, limited knowledge about the coronavirus or COVID-19 disease, and lack of trust in physicians and hospitals variables. The Odds Ratio (OR) and 95% confidence interval (CI) were reported for all variables. Receiver operating characteristic (ROC) curve was used to evaluate the risk prediction of the model (19). The Statistical Package for the Social Sciences (SPSS), version 20.0, for Windows and STATA, version 11 were used for the analyses. The tests were two-tailed and *P* < 0.05 was considered to indicate statistical significance.

## Results

### Piloting and Validation

The initial survey was distributed to 100 individuals. At least a week later, the same survey was distributed to 50 individuals from the same group in order to examine the validity and reproducibility of the survey. Analyses of convergent validity revealed that all items in all sections significantly correlated with the total score (*P* < 0.001) except for one statement in the “perceptions toward COVID-19” section. The statement was “if a vaccine is made available, it should be mandatory for all to take it.” This statement was deleted in the final version of the questionnaire.

Analyses of divergent validity revealed that the total scores of “knowledge of clinical trials” significantly correlated with “motivating factors toward participation in COVID-19 clinical trials” (*r* = 0.31, *P* = 0.004), the total scores of “perceptions toward COVID-19” section correlated with “attitude toward self-participation or participation of a family member in COVID-19 vaccine or drug clinical trials” (*r* = 0.30, *P* = 0.005), and there was an inverse correlation between “motivating factors toward participation in COVID-19 clinical trials” and “deterring factors of participation in a COVID-19 clinical trial” (*r* = −0.29, *P* = 0.007).

Reliability analyses revealed acceptable Cronbach's α scores and ICC for all sections. The score for the “knowledge of clinical trials” section had a Cronbach's α of 0.70 and ICC ranged between 0.62 and 0.75, the “perceptions toward COVID-19” section had a Cronbach's α score of 0.72 and ICC ranged between 0.65 and 0.70, the “motivating factors toward participation in COVID-19 clinical trials” section had a Cronbach's α of 0.83 and ICC ranged between 0.60 and 0.80, the “deterring factors of participation in a COVID-19 clinical trial” had a Cronbach's α of 0.85 and ICC ranged between 0.63 and 0.88, and, finally, the “attitude toward self-participation or participation of a family member in COVID-19 vaccine or drug clinical trials” had a Cronbach's α of 0.89 and ICC ranged between 0.64 and 0.86.

### Characteristics of Participants

**Figure 1:**
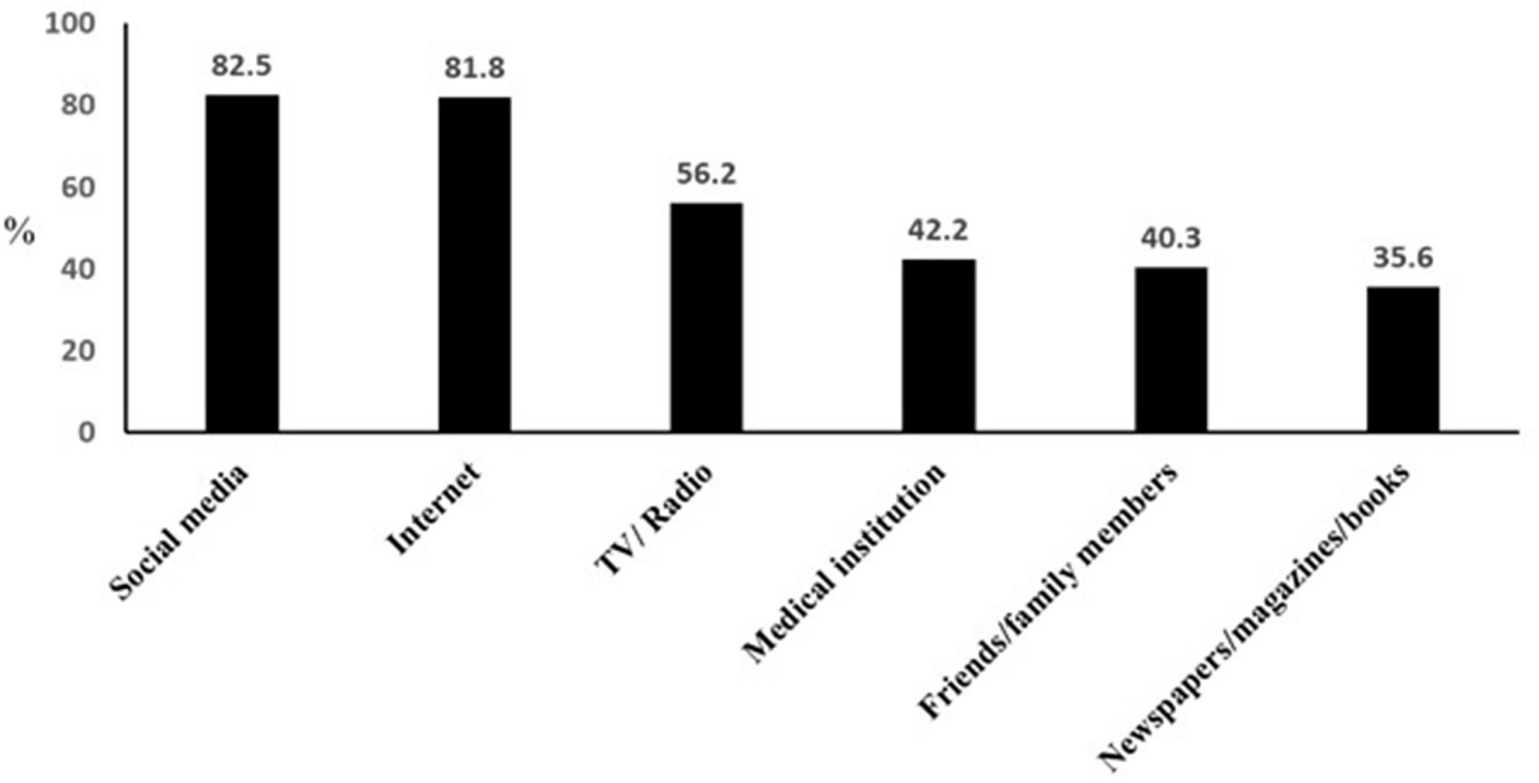
Sources of knowledge regarding clinical trials.

Fifteen hundred and seventy-six individuals participated in the study. Table 1 summarizes the demographic characteristics of the study population. More than half of them were from KSA (53.5%), followed by Egypt (28.4%), then Jordan (18.1%). About two-thirds (64.4%) of the study population aged < 40 years, and 58% of them were males. The majority (82.1%) resided in urban areas and 61.4% had a diploma or a bachelor's degree, whereas, a quarter held a higher degree. Almost half of them (49.6%) thought they were infected with the coronavirus, but only 6.3% of them were, and 16.7% were in contact with an actual COVID-19 patient. The majority of the study population (80.3%) indicated that they did not suffer from chronic diseases. Interestingly, three quarters (74.9%) of respondents were knowledgeable of the term “clinical trials” prior to the survey. The main source of information of clinical trials was obtained from social media (82.5%) and internet search (81.8%), followed by TV/radio, a medical institute, or from family or friends (Figure 1).

**Table 1:** Baseline data of participants and association with knowledge of clinical trials.

**Variables**	**No. of respondents (%) (*N* = 1,576)**	**Attitude toward participating in a clinical trial**		**Rho (*P*-value)**	*P*-value^*^
		**Positive (*n* = 909)**	**Negative (*n* = 667)**		
**Country**					
Egypt	448 (28.4)	256 (28.2)	192 (28.8)	**0.11 (<0.001)** ^**^	**0.001**
KSA	843 (53.5)	516 (56.8)	327 (49.0)		
Jordan	285 (18.1)	137 (15.1)	148 (22.2)		
**Gender**					
Male	907 (58)	568 (63)	339 (51.1)	**0.16 (<0.001)**	**0.001**
Female	658 (42)	334 (37)	324 (48.9)		
**Age categories**					
18–29	403 (25.6)	245 (27.0)	158 (23.7)	**−0.07 (0.004)**	0.46
30–39	611 (38.8)	351 (38.6)	260 (39.0)		
40–49	353 (22.4)	190 (20.9)	163 (24.4)		
50–59	155 (9.8)	92 (10.1)	63 (9.4)		
60–69	45 (2.9)	27 (3.0)	18 (2.7)		
>=70	9 (0.6)	4 (0.4)	5 (0.7)		
**Residence**					
Urban	1,287 (82.1)	727 (80.3)	560 (84.5)	**0.08 (0.001)**	**0.04**
Rural	281 (17.9)	178 (19.7)	103 (15.5)		
**Education**					
Elementary	4 (0.3)	2 (0.2)	2 (0.3)	**–**0.04 (0.21)	0.63
Preparatory	22 (1.4)	16 (1.8)	6 (0.9)		
High school	183 (11.7)	107 (11.8)	76 (11.5)		
Diploma/Bachelor degree	962 (61.4)	557 (61.5)	405 (61.1)		
High diploma/Master/PhD	397 (25.3)	223 (24.6)	174 (26.2)		
**Monthly income**					
<500 USD	189 (13.3)	108 (12.9)	81 (13.9)	**−0.08 (0.002)**	0.07
500–1,000 USD	428 (30.2)	265 (31.8)	163 (27.9)		
1,000–1,500 USD	319 (22.5)	200 (24.0)	119 (20.4)		
1,500–2,000 USD	188 (13.3)	99 (11.9)	89 (15.2)		
>2,000 USD	294 (20.7)	162 (19.4)	132 (22.6)		
**Ever diagnosed with COVID-19**					
Yes	99 (6.3)	59 (6.5)	40 (6.0)	**–**0.02 (0.35)	0.79
No	1,289 (82.0)	748 (82.3)	541 (81.7)		
Not sure	183 (11.6)	102 (11.2)	81 (12.2)		
**Contact with a COVID-19 case**					
Yes	262 (16.7)	161 (17.7)	101 (15.2)	**–**0.03 (0.21)	0.25
No	1,153 (73.4)	664 (73.1)	489 (73.8)		
Not sure	156 (9.9)	83 (9.1)	73 (11.0)		
**Suspected of having COVID-19**					
Yes	779 (49.6)	460 (50.7)	319 (48.0)	−0.02 (0.39)	0.59
No	661 (42.0)	373 (41.1)	288 (43.4)		
Not sure	132 (8.4)	75 (8.3)	57 (8.6)		
**History of chronic disease(s)**					
Yes	309 (19.7)	182 (20.0)	127 (19.0)	0.004 (0.87)	0.25
No	1,276 (80.3)	727(80.0)	540 (81.0)		
**Heard about clinical trial s before**					
Yes	1,174 (74.9)	680 (75.1)	494 (74.5)	0.002 (0.98)	0.78
No	402 (25.1)	229 (24.9)	173 (25.5)		

* *This P-value is based on Chi-square test*.

** * Bold values indicate significant P values (<0.05)*.

### Willingness to Participate in COVID-19 Clinical Trials

**Figure 2:**
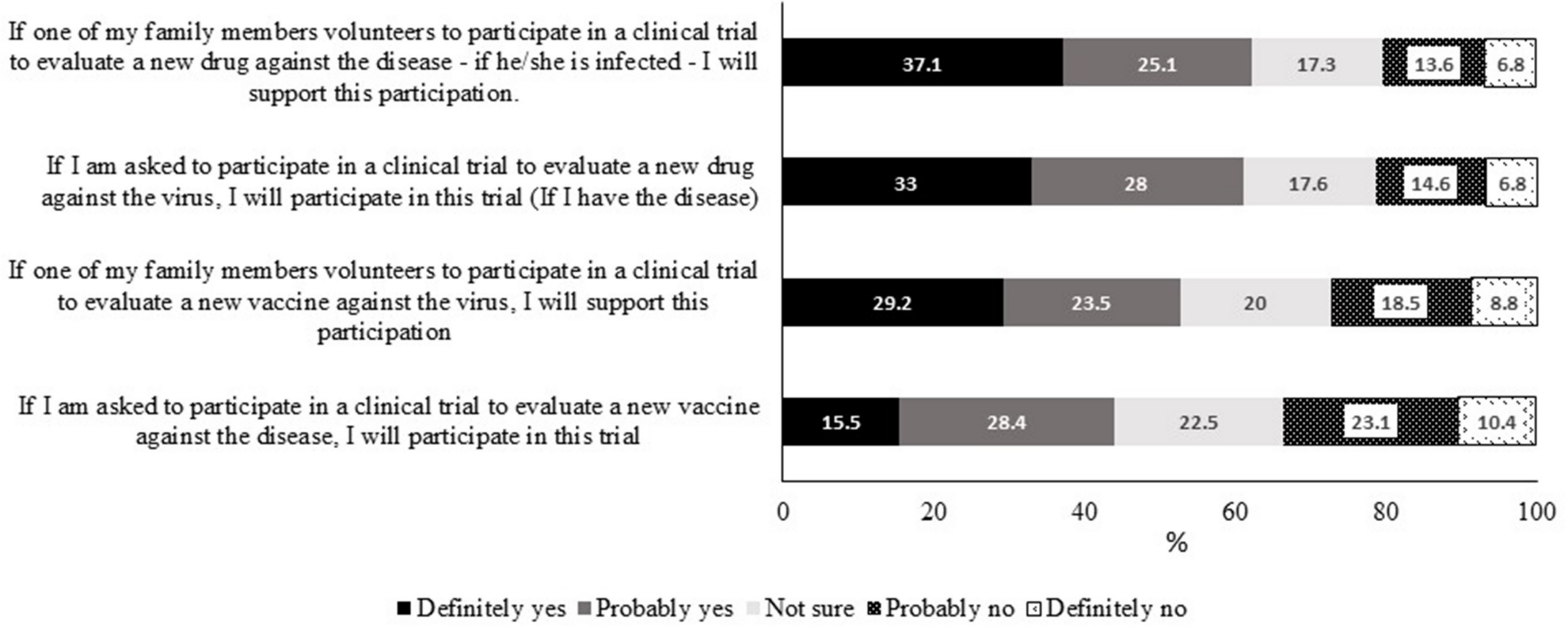
Attitudes of participants toward self-participation or participation of a family member in COVID-19 vaccine or drug clinical trials.

Respondents were asked about their willingness to participate in COVID-19 clinical trials involving either a vaccine or a drug and their attitude if a family member expressed willingness to participate in such trials. Over half of them (57.6%) had an overall positive attitude. More specifically, ~60% of respondents indicated they would either definitely or probably participate in a drug clinical trial (Figure 2). This positive attitude dropped by 16% toward participating in a vaccine trial whereby about 43.9% either definitely or probably participate in a clinical trial for a vaccine. The decrease in willingness came specifically from those who were “definite” participants who were 33% of respondents for a drug trial vs. 15.5% for a vaccine trial. The difference in supporting the participation of a family member in a vaccine trial vs. a drug trial (52.7 vs. 62.2%, respectively) was also observed. Interestingly, more respondents were hesitant toward vaccine trials compared with drug trials.

Attitudes of participants were divided into either positive or negative based on the scoring system (see Methodology) and were associated with sociodemographic characteristics (Table 1). Participants with negative attitudes were females, living in urban areas and from Jordan compared to those with positive attitudes. Those from KSA appeared to have a more positive attitude. However, it is important to mention that two-thirds of respondents from Jordan were females, whereas females were one-third of KSA respondents (data not shown). The total attitude score positively correlated with the country and residence and negatively correlated with age and gender (Table 1).

### Perceptions of COVID-19 and Its Association With Willingness to Participate in COVID-19 Clinical Trials

Respondents were asked whether they agree with several statements related to COVID-19 (Table 2). Respondents had very good knowledge that clinical trials are conducted to ensure vaccine or drug safety (93.4%) and that they are initially conducted on animals (86.3%). However, ~60% indicated that they would not take the vaccine or drug unless they are sure of their efficacy. Interestingly, 28.5% of respondents thought that participation in a vaccine clinical trial might cause them to be affected by COVID-19. More respondents were concerned that a family member would be affected by COVID-19 than themselves (85.6 vs. 62.3%, respectively). In addition, more than half of the respondents indicated that any new vaccine or drug for COVID-19 will be exploited either commercially (65%) or politically (62.8%), and thought that price of a vaccine or a drug would not be reasonable to the public (60 and 58.7%, respectively). Nearly, one-third of the respondents believed that there was exaggerated attention to this virus. Only a small portion (9.2%) of respondents thought that COVID-19 is linked to death.

**Table 2:** Perceptions of respondents on COVID-19 and correlation with attitude toward participation in COVID-19 clinical trials.

**Do you agree with the following statements? (Yes/no)**	**No. of “yes” respondents of the total sample (%) (*N* = 1,576)**	**Attitude toward participating in a clinical trial**		**Rho (*P*-value)**	*P*-value^*^
		**Positive (%) (*n* = 909)**	**Negative (%) (*n* = 667)**		
The goal of conducting a clinical trial on a vaccine or drug against COVID-19 is to determine its safety and efficacy in humans.	1,464 (93.4)	859 (94.8)	605 (91.4)	**0.06 (0.02)** ^**^	**0.01**
Before conducting a clinical trial on either a drug or a vaccine for COVID-19, its safety and effectiveness should first be tested in animals to ensure their safety in humans.	1,352 (86.3)	784 (86.8)	568 (85.7)	−0.01 (0.71)	0.51
When a vaccine for COVID-19 becomes available, I will not take it unless I am sure it is effective.	969 (61.9)	524 (57.8)	445 (67.5)	**0.16 (<0.001)**	** <0.001**
When a drug for COVID-19 becomes available, I will not take it unless I am sure it is effective.	925 (59.1)	493 (54.5)	432 (65.5)	–**0.15 (<0.001)**	** <0.001**
Participating in a clinical trial on a vaccine for COVID-19 will increase my risk of contracting it.	447 (28.5)	217 (24.0)	230 (34.7)	–**0.14 (<0.001)**	** <0.001**
I am worried that I will contract COVID-19.	978 (62.3)	564 (62.2)	414 (62.4)	−0.003 (0.31)	0.92
I am worried a family member (s) would contract COVID-19.	1,340 (85.6)	779 (86.0)	561 (85.0)	−0.02 (0.43)	0.59
A newly developed vaccine or drug for COVID-19 will be exploited commercially.	1,017 (65.0)	567 (62.7)	450 (68.2)	–**0.08 (0.001)**	**0.02**
A newly developed vaccine or drug for COVID-19 will be exploited politically.	983 (62.8)	559 (61.6)	424 (64.4)	–**0.05 (0.04)**	0.26
Once a treatment for COVID-19 is made available, it will be affordable for most.	647 (41.3)	409 (45.1)	238 (36.1)	**0.08 (0.002)**	** <0.001**
Once a vaccine for COVID-19 is made available, it will be affordable for most.	627 (40.0)	392 (43.2)	235 (35.7)	**0.08 (0.001)**	**0.003**
Interest in COVID-19 is exaggerated in general.	563 (35.9)	318 (35.1)	245 (37.1)	−0.01 (0.60)	0.40
Contracting COVID-19 is closely linked to death.	144 (9.2)	90 (9.9)	54 (8.2)	**0.05 (0.03)**	0.23

* *P-value is based on Chi-square test*.

** * Bold values indicate significant P values (< 0.05)*.

We tested the association between their perceptions and attitudes toward clinical trials. Several perceptions were found to influence participants' willingness to participate in COVID-19 clinical trials. Fear of an increased risk of infection with the virus, potential commercial exploitation through excessive pricing, and issues related to drug or vaccine efficacy were all found to significantly associate with negative attitudes toward participation in COVID-19 clinical trials.

### Factors Influencing the Respondents' Willingness to Participate in COVID-19 Clinical Trials

Respondents were given statements that had either positive or negative connotations to examine their decision to participate in clinical trials (Table 3). Contribution to protecting family was the most selected motivating statement (80.5%). This was followed by conducting trials in accordance with scientific and ethical guidelines (77.1%). In addition, three-quarters of the respondents believed that participation could protect the community, restore life to normal, and save humankind. More than half of the respondents (54.3%) indicated that receiving additional healthcare would motivate them to participate in COVID-19 clinical trials, and only 21% would participate if granted financial compensation. All of the motivating factors significantly and directionally correlated with the positive attitude score toward participation in COVID-19 clinical trials with correlation coefficients of 0.40 and higher except for gaining benefits having correlation coefficients of 0.31 and of 0.13 for receiving healthcare benefits and financial compensation, respectively. All positive statements showed significant associations with the attitude toward participation in COVID-19 clinical trials (*P* <0.001).

**Table 3:** Factors influencing decision to participate in COVID-19 clinical trials.

**Would the following statements influence your decision to participate in a COVID-19 clinical trial?**	**No. of “yes” respondents of the total sample (%) (*N* = 1,576)**	**Attitude toward participating in a clinical trial**		**Rho (*P*-value)**	*P*-value^*^
		**Positive (%) (*n* = 909)**	**Negative (%) (*n* = 667)**		
**A. Positive factors**					
Contributing to the protection of my family from COVID-19 encourages me to participate.	1,259 (80.5)	836 (92.4)	423 (64.2)	**0.40 (<0.001)** ^**^	** <0.001**
The conduct of clinical trials will be in accordance with the scientific research, and ethical guidelines encourages me to participate.	1,202 (77.1)	825 (91.0)	377 (57.6)	**0.45 (<0.001)**	** <0.001**
Contributing to the return of normal life encourages me to participate.	1,187 (76.2)	810 (89.6)	377 (57.7)	**0.43 (<0.001)**	** <0.001**
Contributing to the salvation of humankind from COVID-19 encourages me to participate.	1,169 (74.9)	803 (88.5)	366 (56.0)	**0.43 (<0.001)**	** <0.001**
Contributing to the protection of my community from COVID-19 encourages me to participate.	1,151 (73.6)	794 (87.7	357 (54.2)	**0.44 (<0.001)**	** <0.001**
Receiving additional health care benefits encourages me to participate.	850 (54.3)	599 (66.1)	251 (38.1)	**0.31 (<0.001)**	** <0.001**
Receiving a financial reward encourages me to participate.	323 (20.6)	216 (23.9)	107 (16.2)	**0.13 (<0.001)**	** <0.001**
**B. Negative factors**					
The probability of occurrence of negative consequences to my health prevents me from participating in such studies.	1,054 (67.4)	528 (58.2)	526 (80.1)	**−0.33 (<0.001)**	** <0.001**
Having limited information about clinical trials, in general, prevents me from participating in such studies.	876 (56.5)	481 (53.3)	395 (61.0)	**−0.13 (<0.001)**	**0.003**
The possibility that these studies will not be conducted in an ethical manner that follows the required scientific and research methods prevents me from participating in such studies.	849 (54.5)	462 (51.2)	387 (59.1)	**−0.11 (<0.001)**	**0.002**
The possibility of exploiting me and turning into a “lab rat” prevents me from participating in such studies.	842 (53.9)	416 (45.9)	426 (65.0)	**−0.24 (<0.001)**	** <0.001**
Lack of trust in pharmaceutical companies, in general, prevents me from participating in such studies.	709 (45.5)	364 (40.3)	345 (52.8)	**−0.17 (<0.001)**	** <0.001**
Having limited information about the novel coronavirus and COVID-19 prevents me from participating in such studies.	684 (44.0)	343 (38.0)	341 (52.4)	**−0.15 (<0.001)**	** <0.001**
The possibility of violating my privacy (such as if my samples and health data are sent to other centers and countries) prevents me from participating in such studies.	558 (35.7)	288 (31.8)	270 (41.1)	**−0.13 (<0.001)**	** <0.001**
Having limited time prevents me from participating in such studies.	525 (34.0)	295 (32.9)	230 (35.5)	**–**0.05 (0.06)	0.28
My family's attitude toward participation in these studies prevents me from participating in such studies.	523 (33.7)	277 (30.7)	246 (37.8)	**−0.09 (<0.001)**	**0.003**
My current medical problems prevent me from participating in such studies.	425 (27.5)	224 (24.9)	201 (30.9)	**−0.07 (0.008)**	**0.009**
Lack of trust in physicians and hospitals, in general, prevents me from participating in such studies.	424 (27.2)	186 (20.6)	238 (36.4)	**−0.19 (<0.001)**	** <0.001**
Lack of trust in researchers and scientists, in general, prevents me from participating in such studies.	321 (20.7)	142 (15.7)	179 (27.5)	**−0.15 (<0.001)**	** <0.001**
Lack of conviction about the value and benefits of these trials prevents me from participating in such studies.	283 (18.3)	135 (15.0)	148 (22.9)	**−0.10 (<0.001)**	** <0.001**
My religious beliefs toward participation in these studies prevent me from participating in such studies.	156 (10.0)	86 (9.5)	70 (10.7)	**–**0.01 (0.58)	0.46
Customs and traditions in my community prevent me from participating in such studies	151 (9.7)	75 (8.3)	76 (11.7)	**–**0.04 (0.09)	**0.03**

* *This P-value is based on Chi-square test*.

** * Bold values indicate significant P values (<0.05)*.

Similarly, several negative statements were provided to respondents, and association with attitude to participate in COVID-19 clinical trials was assessed. Fear of negative health consequences was found to be the main hindering factor to participate in clinical trials. The latter was indicated by 67.4% of respondents and had the most negative correlation (*r* = −0.33, *P* < 0.001) with a significance of <0.001 between those with positive vs. negative attitudes. Interestingly, lack of knowledge of clinical trials was the second highest factor that negatively influenced participation in COVID-19 clinical trials. It was selected by 56.5% of the respondents and significantly correlated with the negative attitude toward participation in clinical trials (*r* = −0.13, *P* < 0.001). Violating research ethics or fear of turning into experimental animals were also considered significant hindering factors with correlation to negative attitudes for almost half of the respondents. Lack of trust in pharmaceutical companies (45.5%), a healthcare system in the form of physicians and hospitals (27.2%) as well as scientists/researchers (20.7%) could prevent respondents from participating in clinical trials. All three statements related to trust significantly correlated with the negative attitudes toward participation in COVID-19 clinical trials. The least factors that might prevent respondents from participating in clinical trials were religious beliefs (10%) and community customs and traditions (9.7%). The latter factors, in addition to having limited time, did not correlate to the attitude toward participation in COVID-19 clinical trials. All negative statements were significantly associated with the attitude toward participation in COVID-19 clinical trials (*P* < 0.05), except for “having limited time prevents me from participation in such studies” and “my religious beliefs toward participation in these studies prevent me from participation in such studies.”

### Predictors of Attitudes Toward Participation in COVID-19 Clinical Trials

**Figure 3:**
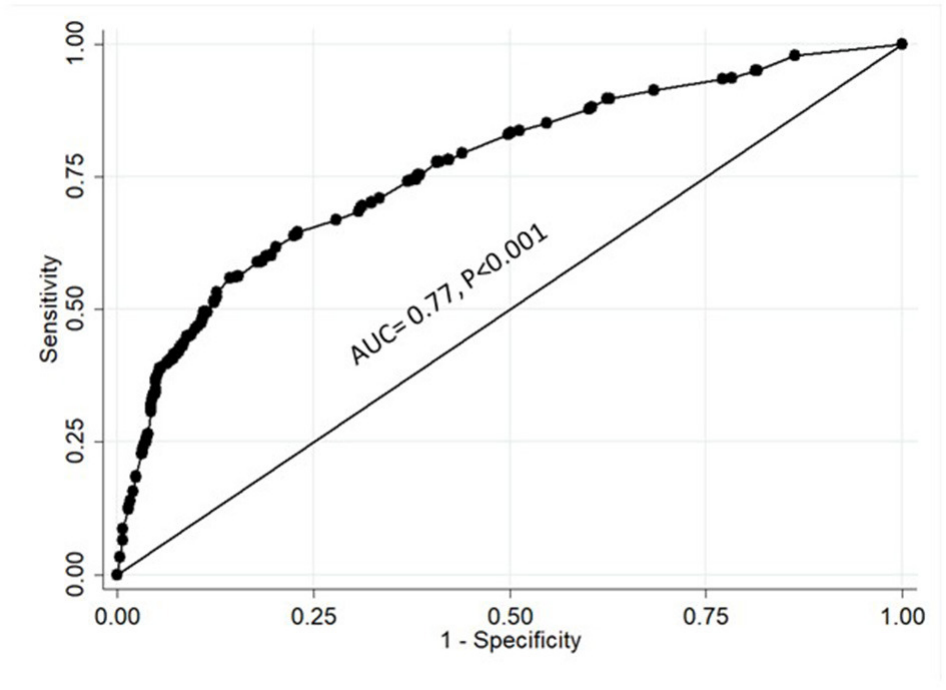
ROC curve analysis for evaluating the prediction of logistic regression model.

A regression analysis of all the statements revealed that, in addition to gender, seven statements were found to predict willingness to participate in COVID-19 clinical trials, four positive predictors, and three negative ones (Table 4). The conduct of clinical trials in accordance with the scientific research and ethical guidelines strongly decreased the risk of not participating in clinical trials (*P* < 0.001). Other factors with positive influence included protection of family from COVID-19 (*P* = 0.007), contribution to return to normal community life (*P* = 0.04), and receiving additional healthcare benefits (*P* < 0.001). On the other hand, the thought that clinical trials can have a negative impact on the health of participants increased the risk of having a negative attitude toward participation in such trials (*P* < 0.001). This was followed by having limited information about the novel coronavirus and COVID-19 (*P* < 0.001) and a lack of trust in physicians and hospitals (*P* = 0.006). Being a female also significantly increased the risk of not participating in COVID-19 clinical trials (*P* = 0.005). It is notable that the country of origin, which correlated with a negative attitude toward participating in COVID-19 clinical trials, was not a predictor as displayed in Table 1. Figure 3 showed that the area under the curve (AUC) was 0.77 which reflects that the model was capable of predicting the attitude of participants toward self-participation or participation of a family member in COVID-19 vaccine or drug clinical trials by 77% (AUC = 0.77, *P* < 0.001).

**Table 4:** Predictors of attitude toward participation in COVID-19 clinical trials.

**Statement**	**Unit of increase**	**OR (95% CI)**	***P*-value**
The conduct of clinical trials will be in accordance with scientific research and ethical guidelines.	No = 0, Yes = 1	0.35 (0.24–0.51)	<0.001
Contributing to the protection of my family from COVID-19.	No = 0, Yes = 1	0.53 (0.33–0.84)	0.007
Receiving additional healthcare benefits.	No = 0, Yes = 1	0.61 (0.47–0.79)	<0.001
Contributing to the protection of my community from COVID-19.	No = 0, Yes = 1	0.65 (0.43–0.98)	0.04
The possibility of getting ill prevents me from participating in such trials.	No = 0, Yes = 1	1.68 (1.26–2.24)	<0.001
Limited knowledge about the coronavirus or COVID-19 disease.	No = 0, Yes = 1	1.58 (1.22–2.04)	<0.001
Lack of trust in physicians and hospitals	No = 0, Yes = 1	1.49 (1.12–1.97)	0.006
Gender	Male = 0, Female = 1	1.42 (1.11–1.81)	0.005

## Discussion

During the first two decades of the twenty-first century, the human race witnessed the emergence of three previously unknown coronaviruses: severe acute respiratory syndrome coronavirus (SARS-CoV), Middle East respiratory syndrome coronavirus (MERS-CoV), and, recently, SARS-CoV-2. Although SARS-CoV-2 is genetically related to SARS-CoV, the new virus has unique features that contributed to its rapid spread globally (20). Although some vaccines and drugs have been approved for COVID-19, significant efforts are still ongoing to support the development of more vaccines and therapeutic drugs. The success of these studies depends on the active engagement of potential participants. In our study, we report that participants from the three countries had a positive attitude toward participation in COVID-19 clinical trials, and this attitude was significantly associated with altruism, personal and community benefits, and conducting the trials according to ethical guidelines. On the other hand, the female gender, lack of trust in physicians and hospitals, and potential negative health consequences were associated with negative attitudes toward participation in these trials.

About three-quarters of the respondents had previous knowledge of clinical trials. This percentage is much higher than previous results reported in Jordan (21.8%) (21) and in Oman (31.3%) (22). On the other hand, they are comparable to the results reported in the United States, where 66% of the participants reported that they had previous information about clinical trials (23). This can be interpreted to the higher knowledge among our participants compared to previous studies in Arab countries as the vast majority of our respondents had, at least, a university degree. In fact, knowledge about clinical trials was associated with higher education in the studies conducted in Jordan and the United States (21, 23). Another reason is the unprecedented media coverage of this pandemic and the news covering clinical trials launched to test new vaccines or treatments for the virus. This has increased public knowledge of clinical trials.

Several platforms represented the sources of information about clinical trials for our participants. Social media and the internet ranked first followed by other platforms. The internet and social media were also the main sources of general information about COVID-19 among the public in Egypt (24). These results are in accordance with a previous population-based survey conducted in Jordan where the internet was the most searched source of health-related information (25). Social media have also been effectively used to communicate research concepts with specific target groups (26). Although the internet and social media provide easy and quick access to information, they can be a source of misinformation, and the public should be educated about their use.

When investigating the attitude toward participation in COVID-19 clinical trials, notable and interesting differences could be observed in regards to two items: first, participation in a vaccine trial vs. a drug trial and, second, personal participation vs. supporting the participation of a family member in a clinical trial. The difference in enthusiasm was more apparent in the percentage of individuals responding with the “very likely” option. This is expected since participation in a COVID-19 drug trial is conditioned by being affected by the virus as stated in the questionnaire and lack of the therapeutic drug. These results may suggest that there are issues associated with vaccines including the concern about the potential association between known vaccines and the development of disease conditions such as autism (27). The overall positive attitudes toward participation in clinical trials and the lack of difference between personal participation in a drug trial or supporting a family member to participate in such trials positively reflect the importance of clinical trials among Arabs.

Our results are comparable to previous reports. In Oman, 50% of participants showed interest in participating in clinical trials related to their medical condition (22). In addition, 58% of KSA respondents in an independent study were willing to participate in a clinical trial if they were healthy (28). However, this was more than twice the percentage of respondents who indicated their willingness to participate in clinical trials in Jordan (21).

Several factors were considered as the predictors of likeliness to participate in COVID-19 clinical trials. Altruism appears to be one factor where respondents indicated that they would participate in clinical trials to protect their families and to return their communities to normal conditions. This is similar to previous studies in three Arab countries, KSA, Egypt, and Qatar, where participation in clinical trials and research is considered a form of charity and means to help society, advance medical knowledge, and help others (28–30). Altruism and hope for a better treatment were the main factors that motivated most cancer patients to participate in oncology clinical trials (6, 31). In general, altruism improves self-image and the sense of fulfillment and usefulness of participants (32).

Previously, a review of factors affecting patients' participation in clinical trials identified personal gain in the form of better healthcare and extra medical attention as the primary reasons for participating in this type of studies (33). Herein, receiving additional healthcare, but not financial reward, was a significant predictor of participation. The same was reported in Qatar where additional medical care was among the factors that encouraged individuals to participate in different types of medical research (30). It seems that both personal and community benefits represent two important motives for participation in clinical trials. These benefits should be clarified to potential participants and can be used to encourage them to share in these studies.

In the introductory section of our questionnaire, the main ethical issues linked to clinical trials were briefly explained in the informed consent. Interestingly, our respondents were aware of the importance of this issue where the conduct of research under ethical guidelines was associated with a positive attitude toward participation and a predictor of participation. Moreover, they had concerns regarding their potential exploitation, being used as “lab rats,” and the potential violation of privacy, all of which were associated with negative attitudes toward participation. A recent study highlighted the presence of racial disparity in COVID-19 clinical trials in the United States and called for justice and equitable selection of participants together with a presentation of demographic data and outcomes of these studies (34). In a previous study in KSA, <50% of participants believed that clinical trials are conducted ethically (28). On the other hand, positive outcomes for self and others, and ethical conduct of different types of research in Qatar encouraged them to join future research initiatives (30). We believe that transparency and assurances to adherence to Good Clinical Practice (GCP) are important factors to encourage participation in clinical trials in Arab countries (35).

Questions arise with the development of vaccines and drugs for COVID-19. One important question is what if a vaccine and or drug is exploited commercially or politically. Recently, the Russian president announced that a locally developed vaccine has been given regulatory approval and could be available to the public soon. As soon as the news spread about the approved vaccine, a debate started about its safety, efficacy, cost, and economics, as well as political implications of this announcement (36–38). It should be noted that the contradictory information in the media may affect public trust in clinical trials and medical research in general. About two-thirds of our respondents were concerned about the commercial and political exploitation of newly developed vaccines or drugs once developed.

The possibility of commercial exploitation has a significant association with the refusal to participate in COVID-19 clinical trials. If participants are convinced that a medical intervention to treat COVID-19 is available at an affordable price; this could encourage them to participate in clinical trials. We call for global collaboration among nations, organizations, and commercial entities to overcome this unprecedented pandemic. Technology transfer is one way to ensure sufficient supplies of vaccines in developing countries. To reach this goal, WHO recommends the achievement of a win-win situation through a commitment from governments to support this kind of technology transfer or the presence of a large local or regional market (39). During the current pandemic, WHO launched the Access to COVID-19 Tools (ACT) Accelerator, which brings together governments and organizations to support the development and fair distribution of diagnostics, treatments, and vaccines needed by different countries in the world (40). Lack of knowledge of two issues is associated with less enthusiasm to participate in clinical trials. One issue is related to the perception that participation in clinical trials can pose a threat to participants' health. The same perception was also reported as the major reason for unwillingness to participate in clinical trials in Jordan (21). Fear of negative consequences on health was emphasized among African Americans in two independent studies (41, 42), and among Danish participants (43). Fear from negative consequences of participation may explain the general negative attitude toward participation among females in our study where they may tend to be more concerned about their families during such pandemic. In fact, Jordanian participants had a negative attitude toward participation in COVID-19 clinical trials compared to KSA participants as most Jordanian respondents were females. This was corrected in the prediction analysis where the country was not a predictor of participation. We believe that this is an appropriate time to increase public awareness of clinical trials and enforce the introduction of this concept into education curricula.

The other knowledge-based issue is the lack of information regarding coronavirus and COVID-19. However, it is not clear what information our respondents exactly need. The media was flooded with news of the virus and the disease. The problem may be due to the contradictory information that the media transmit regarding the virus, the mechanisms of transmission, and the consequence of infection. These could result in building doubts about the disease and its severity and, hence, make people hesitant about participating in a trial. Conflicting information can also create mistrust in the healthcare system including physicians and pharmaceutical companies. What is interesting is the association of low trust in physicians and hospitals in discouraging participation in clinical trials. About three-quarters of KSA respondents in a previous study were willing to participate in clinical trials after discussing this issue with their family physician (28). The intentions of physicians, when offering the public the opportunity to participate in a clinical trial, can be sensed and can affect their decisions (33). The sense of trust can be divided into four dimensions: general trustworthiness, perceptions of discrimination, deception, and exploitation (44). A scale to measure trust was developed (45) and it would be interesting to modify it, taking into consideration the different cultural backgrounds in the Arab world, and apply it in an independent study.

It is promising that although several negative statements were found to correlate with the unwillingness to participate in clinical trials, they were not predictors. One example is the thought that a vaccine or drug will be exploited commercially. Another is the possibility of turning those enrolled in clinical trials into “lab rats.”

## Conclusions and Recommendations

In general, Arab citizens have good knowledge of and a positive attitude toward COVID-19 clinical trials. It is recommended to increase public awareness of clinical trials and the significance of diversifying participation using various means. We recommend further studies to understand the factors that may affect trust among citizens in the Arab region, and how these factors influence participation in research in general and, specifically, clinical trials. The role of physicians in increasing awareness and trust is critical and should be emphasized in any educational initiative. Fair distribution of benefits between high- and low-income countries, especially when it comes to the COVID-19 vaccine or treatment, is an important strategy to overcome this pandemic. Clear international policies about these issues should be discussed and communicated with the public to encourage their participation in research regarding this global problem.

### Limitations of the Study

In light of the limited studies related to the topic, the results of our study add to the global evidence about the perception and attitudes of Arab citizens in participating in clinical trials and, particularly, those that target COVID-19. Using multiple country sampling and settings and the large sample size contribute to the validity as well as the generalizability of the study findings. A major strength of this study is the inclusion of three countries that represent a diverse group of Arab peoples thus providing credibility to the data. However, there are also some limitations of the study that must be considered. First, data are based on a self-reporting, electronic questionnaire; this is a method that could jeopardize participants' understanding of some items or may allow them to answer the questionnaire hastily. Additionally, using online data collection platforms could have prevented us from reaching a certain segment of populations of the three societies, i.e., those with lower education or lower income. We tried to overcome this limitation by using multiple platforms.

## Data Availability Statement

The raw data supporting the conclusions of this article will be made available by the authors, without undue reservation.

## Ethics Statement

The studies involving human participants were reviewed and approved by Institutional Review Board, Jordan University Hospital, The University of Jordan, Institutional Review Board, King Fahad Medical City, and Institutional Review Board, National Cancer Institute, Cairo University. Written informed consent for participation was not required for this study in accordance with the national legislation and the institutional requirements.

## Author Contributions

AA proposed the idea, participated in data collection, and wrote the manuscript. SA analyzed data, participated in data collection, and manuscript writing. MK led data collection from KSA and revised the manuscript. MS, BA, RS, MAl, and FA participated in data collection and revised the manuscript. MAh supervised and led the study, participated in data collection, and wrote the manuscript. All authors contributed to the article and approved the submitted version.

## Conflict of Interest

The authors declare that the research was conducted in the absence of any commercial or financial relationships that could be construed as a potential conflict of interest.
