# Corrigendum: Factors influencing participation in COVID-19 clinical trials: a multinational study

**DOI:** 10.3389/fmed.2023.1230259

**Published:** 2023-06-22

**Authors:** Ahmed Samir Abdelhafiz, Samar Abd ElHafeez, Mohammad Adnan Khalil, Manal Shahrouri, Bandar Alosaimi, Raneem O. Salem, Mohamed Alorabi, Fatma Abdelgawad, Mamoun Ahram

**Affiliations:** ^1^Department of Clinical Pathology, National Cancer Institute, Cairo University, Cairo, Egypt; ^2^Epidemiology Department, High Institute of Public Health, Alexandria University, Alexandria, Egypt; ^3^Department of Basic Medical Sciences, Faculty of Medicine, King Fahad Medical City, Riyadh, Saudi Arabia; ^4^Office of Monitoring, Research, Learning and Evaluation, Tetra Tech DPK, Amman, Jordan; ^5^Department of Research Labs, Research Center, King Fahad Medical City, Riyadh, Saudi Arabia; ^6^Department of Clinical Oncology, Faculty of Medicine, Ain Shams University, Cairo, Egypt; ^7^Department of Pediatric Dentistry and Dental Public Health, Faculty of Dentistry, Cairo University, Cairo, Egypt; ^8^Department of Physiology and Biochemistry, The University of Jordan, Amman, Jordan

**Keywords:** COVID-19, clinical trials, Arabs, bioethics, attitude

In the published article, an author name was incorrectly written as Bandar Alosaim. The correct spelling is Bandar Alosaimi.

The authors apologize for this error and state that this does not change the scientific conclusions of the article in any way. The original article has been updated.

